# Genome-wide patterns of latitudinal differentiation among populations of *Drosophila melanogaster* from North America

**DOI:** 10.1111/j.1365-294X.2012.05731.x

**Published:** 2012-08-22

**Authors:** Daniel K Fabian, Martin Kapun, Viola Nolte, Robert Kofler, Paul S Schmidt, Christian Schlötterer, Thomas Flatt

**Affiliations:** * Institut für Populationsgenetik, Vetmeduni ViennaVeterinärplatz 1, A-1210 Vienna, Austria; † Department of Biology, University of Pennsylvania216 Leidy Laboratories, Philadelphia, PA, 19104 USA; ‡ Wissenschaftskolleg zu Berlin, Institute for Advanced StudyWallotstrasse 19, 14193, Berlin Germany; § Department of Ecology and Evolution, University of LausanneUNIL Sorge CH-1015, Lausanne, Switzerland

**Keywords:** adaptation, cline, *Drosophila melanogaster*, latitudinal variation, pool-seq, single nucleotide polymorphism

## Abstract

Understanding the genetic underpinnings of adaptive change is a fundamental but largely unresolved problem in evolutionary biology. *Drosophila melanogaster*, an ancestrally tropical insect that has spread to temperate regions and become cosmopolitan, offers a powerful opportunity for identifying the molecular polymorphisms underlying clinal adaptation. Here, we use genome-wide next-generation sequencing of DNA pools ('pool-seq') from three populations collected along the North American east coast to examine patterns of latitudinal differentiation. Comparing the genomes of these populations is particularly interesting since they exhibit clinal variation in a number of important life history traits. We find extensive latitudinal differentiation, with many of the most strongly differentiated genes involved in major functional pathways such as the insulin/TOR, ecdysone, torso, EGFR, TGFβ/BMP, JAK/STAT, immunity and circadian rhythm pathways. We observe particularly strong differentiation on chromosome *3R*, especially within the cosmopolitan inversion *In(3R)Payne*, which contains a large number of clinally varying genes. While much of the differentiation might be driven by clinal differences in the frequency of *In(3R)P*, we also identify genes that are likely independent of this inversion. Our results provide genome-wide evidence consistent with pervasive spatially variable selection acting on numerous loci and pathways along the well-known North American cline, with many candidates implicated in life history regulation and exhibiting parallel differentiation along the previously investigated Australian cline.

## Introduction

Unravelling the genetic basis of adaptive change in natural populations is one of the most fundamental aims of evolutionary biology (Dobzhansky 1970; Lewontin 1974; Nielsen 2005; Stinchcombe & Hoekstra 2007; Stapley *et al*. 2010; Barrett & Hoekstra 2011; Rockman 2012). For example, many species that occupy heterogeneous environments across their distributional range are subject to spatially varying selection that may cause genetic differentiation and local adaptation despite gene flow and genetic drift (Levene 1953; Karlin & McGregor 1972a,b; Felsenstein 1976; Endler 1977; Slatkin 1978; Barton 1983; Hedrick 2006). Consequently, much work has focused on identifying polymorphisms that underlie adaptation to spatial heterogeneity and their consequences for quantitative variation (Lewontin & Krakauer 1973; Latta 1998; Le Corre & Kremer 2003; Beaumont & Balding 2004; Hedrick 2006; Whitlock 2008; Nosil *et al*. 2009; Ma *et al*. 2010).

One common approach for identifying polymorphisms that might be targets of spatially variable selection is to search for alleles that show exceptionally strong differentiation among geographically distinct populations: strong outlier 'signals' are taken to be indicative of selection and local adaptation relative to background 'noise' caused by gene flow and drift (Lewontin & Krakauer 1973, 1975; Black *et al*. 2001; Luikart *et al*. 2003; Beaumont 2005; Akey 2009; Akey *et al*. 2010). While this method has been criticized (see discussion in Beaumont 2005), for instance because large genetic differentiation can also be due to demographic factors independent of selection, it has generally proved to be quite robust against effects of demography and thus relatively successful at identifying putatively adaptive loci (Schlötterer 2002; Beaumont & Balding 2004; Beaumont 2005; Stinchcombe & Hoekstra 2007; Nosil *et al*. 2009; Kolaczkowski *et al*. 2011). The outlier method might be especially powerful when applied to situations for which there exists evidence of genetic, phenotypic and ecological adaptation caused by spatially variable selection. Clines, that is, changes in phenotypes and/or allele frequencies along a continuous environmental gradient, offer a particularly promising opportunity in this respect. Because clines are often highly repeatable across different geographical regions, populations and species, both at the level of phenotypic and genetic change, they are widely thought to reflect spatially varying selection (Mayr 1963; Dobzhansky 1970; Endler 1977; Barton 1983, 1999).

The probably most comprehensively studied cases of clinal variation maintained by spatially variable selection are the latitudinal clines observed in the fruit fly, *Drosophila melanogaster* (David & Bocquet 1975; Singh & Rhomberg 1987; Hale & Singh 1991; Hoffmann & Weeks 2007). *Drosophila melanogaster* is an ancestrally tropical species from sub-Saharan Africa that has colonized North America and Australia over the last few 100 years (David & Capy 1988; Lachaise & Silvain 2004), and the establishment of derived populations in temperate regions is thought to have resulted in a number of climatic adaptations (Bouletreau-Merle *et al*. 2003; Hoffmann *et al*. 2003; Sezgin *et al*. 2004; Schmidt *et al*. 2005a,b; Hoffmann & Weeks 2007; Paaby & Schmidt 2009). At the phenotypic level, clinal variation has been documented for a number of major life history traits, including developmental time, body size, ovariole number, fecundity, stress resistance, lifespan, reproductive dormancy and overwintering ability (David & Bocquet 1975; Coyne & Beecham 1987; James & Partridge 1995; Mitrovski & Hoffmann 2001; Hoffmann *et al*. 2002; De Jong & Bochdanovits 2003; Schmidt *et al*. 2005a,b,^2008^; Kennington *et al*. 2007; Schmidt & Paaby 2008). Similarly, at the genetic level, latitudinal clines have been identified for numerous allozyme, DNA and chromosome inversion polymorphisms in both North American and Australian populations (Mettler *et al*. 1977; Knibb 1982; Oakeshott *et al*. 1982; Schmidt *et al*. 2000, 2008; Gockel *et al*. 2001; De Jong & Bochdanovits 2003; Sezgin *et al*. 2004; Anderson *et al*. 2005; Hoffmann & Weeks 2007; Turner *et al*. 2008; Kolaczkowski *et al*. 2011; Paaby *et al*. 2010).

Since many genetic and phenotypic clinal patterns are observed in a parallel fashion on different continents, latitudinal variation is likely to be driven by spatially variable selection, not by demography (Knibb 1982; Singh & Rhomberg 1987; De Jong & Bochdanovits 2003; Turner *et al*. 2008). The notion that clinal variation is mainly due to selection is also consistent with the observation that putatively neutral markers are typically not well correlated with latitude (Hale & Singh 1991; Gockel *et al*. 2002; Kennington *et al*. 2003). However, despite the impressive body of work on clinal variation in *D. melanogaster* and other species, our understanding of the genetic—and in particular the genomic—basis of latitudinal differentiation and adaptation remains incomplete.

Significant progress in uncovering the genetic factors underlying latitudinal differentiation in *D. melanogaster* has recently been made by two studies that characterized clines on a genomic scale (Turner *et al*. 2008; Kolaczkowski *et al*. 2011). Turner *et al*. (2008) used tiling arrays with approximately three million markers to characterize differentiation between northern and southern populations of *D. melanogaster* from the east coast of North America and Australia. The authors identified many interesting genomic regions underlying latitudinal differentiation, including several showing parallel differentiation between the North American and Australian clines. However, the resolution of this study was limited, with one 25-bp array probe for approximately every 40 bp of the genome. More recently, the Australian cline was re-examined with much higher resolution using next-generation sequencing technology by Kolaczkowski *et al*. (2011). By comparing two northern and two southern populations, the authors identified major patterns of clinal differentiation, with strong evidence for selection acting on a number of key biological functions and pathways. However, only the endpoints of the cline were compared, and sequencing coverage was relatively low (8–12 fold).

Here, we aim to complement and extend these recent efforts by characterizing, for the first time, genome–sequence-based patterns of latitudinal differentiation along the well-known North American cline (Oakeshott *et al*. 1982; Coyne & Beecham 1987; Singh & Rhomberg 1987; Hale & Singh 1991; Berry & Kreitman 1993; Schmidt *et al*. 2008; Paaby *et al*. 2010). We apply whole-genome next-generation sequencing, with relatively high sequencing coverage (~45-fold), to DNA pools ('pool-seq') from a northern (Maine), an intermediate (Pennsylvania) and a southern (Florida) population. Describing patterns of genomic differentiation among these populations is particularly interesting since they differ in major life history traits (Schmidt *et al*. 2005a,b; Schmidt & Paaby 2008). Our first objective is thus to generate a comprehensive catalogue of candidate genes and pathways that might underlie life history phenotypes known to vary along the North American cline. Our second goal is to examine the contribution of the major chromosomal inversion *In(3R)Payne* to clinal variation and to identify polymorphisms likely independent of it. Finally, by comparing our results to those of Kolaczkowski *et al*. (2011), we investigate parallel clinal variation between the North American and Australian clines—finding evidence for parallel genetic differentiation between two independent clines would considerably strengthen the case for spatially varying selection acting at specific loci.

## Materials and methods

### Population samples

We analysed three populations from the United States east coast, collected between 2009 and 2010 by P. Schmidt in fruit orchards using aspiration/netting on fallen fruit: (i) a southern population from Florida (F; Fruit and Spice Park, Homestead, FL; 25°32′N, 80°29′W; *n* = 39 isofemale lines; 07/2010); (ii) an intermediate population from Pennsylvania (P; Linvilla Orchards, Media, PA; 39°53′N, 75°24′W; *n* = 102 isofemale lines; 07/2009); and (iii) a northern population from Maine (M; Rocky Ridge Orchard, Bowdoin, ME; 44°1′N, 69°56′W; *n* = 86 isofemale lines; 10/2009) (Fig.). Populations from southern Florida and mid-coastal Maine approximate the southern and northern limits of *D. melanogaster* along the eastern US cline, whereas populations from the mid-Atlantic region (Pennsylvania) are intermediate with regard to climate and life history phenotypes, including the incidence of reproductive dormancy (Schmidt & Paaby 2008).

**Fig 1:**
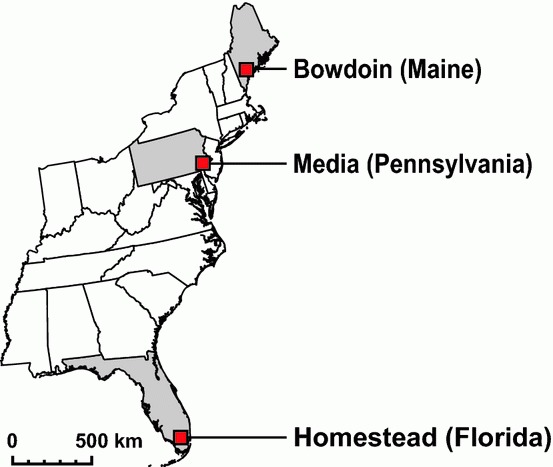
Sampling locations. Three populations were sampled along the United States east coast for genome-wide 'pool-seq': the population from Florida (F) approximates the southern range limit of *Drosophila melanogaster*; the population from the mid-Atlantic region (Pennsylvania, P) is intermediate in terms of climate and the population from Maine (M) approximates the northern range limit. See text for further details.

### Sequencing, mapping and data processing

We used 'pool-seq' to estimate allele frequencies and identify candidate single nucleotide polymorphisms (SNPs) (Futschik & Schlötterer 2010). For each population, we prepared one pooled sample in a single tube, using one female per line (pool sizes: Florida, *n* = 39 females; Pennsylvania, *n* = 102 females; Maine, *n* = 86 females), adjusted the ratio of homogenization buffer to the number of flies in a given pool and homogenized pools with an Ultra-Turrax T10 (IKA-Werke, Staufen, Germany). Genomic DNA was extracted using the Qiagen DNeasy Blood and Tissue Kit (Qiagen, Hilden, Germany). We fragmented genomic DNA using a Covaris S2 device (Covaris, Inc. Woburn, MA, USA) and prepared paired-end genomic libraries (using 5 μg of genomic DNA for each pool and library) with NEBNext DNA Sample Prep modules (New England Biolabs, Ipswich, MA, USA) following the manufacturer's instructions. Cluster amplification was performed using the TruSeq PE Cluster Kit v5 on a cluster station, and each sample was sequenced on one lane of a Genome Analyzer IIx using TruSeq SBS 36 Cycle Kits v5 (Illumina, San Diego, CA, USA). 101 bp paired-end reads were filtered for a minimum average base quality score of 18 and trimmed using PoPoolation (Kofler *et al*. 2011a); only reads with a minimum length > 50 bp after trimming were used for mapping. Trimmed reads were mapped against the FlyBase *D. melanogaster* reference genome r5.40 (http://flybase.org; Adams *et al*. 2000) with the Burrows-Wheeler alignment tool bwa v0.5.8c (Li & Durbin 2009), using the following parameters: -l 150, -n 0.01, -e 12, -d 12 and -o 2. Paired-end data were merged to single files in sam format with the 'sampe' option of bwa. Files were converted to BAM format with SAMtools v0.1.9 (Li *et al*. 2009) and filtered for a minimum mapping quality of 20. BAM files were transformed to pileup files using SAMtools; indels and simple sequence repeats were masked using PoPoolation and RepeatMasker (http://www.repeatmasker.org). RepeatMasker was run using default sensitivity parameters including the search for simple repeats but without searching for bacterial insertion elements (option: -no_is). Mean coverage was highly consistent and uniform among populations and chromosomal arms (Fig. S1, Supporting information); mean sequencing quality (Phred score) was 37 for Maine and 38 for both Florida and Pennsylvania (details not shown). Raw sequencing data prior to trimming and mapping are available as FASTQ files at the Sequence Read Archive (SRA) of the European Nucleotide Archive (ENA) hosted by EMBL-EBI under accession ERP001535 (http://www.ebi.ac.uk/ena/data/view/ERP001535). A detailed description of our bioinformatic analysis pipeline can be found in Appendix S13 (Supporting information).

To identify candidate genes, we used an individual SNP-based rather than a window-based approach, except where indicated otherwise; the latter averages across many sites within a given window and might fail to detect strongly differentiated individual SNPs. The window-based approach could thus possibly be biased towards finding significant differentiation in windows with relatively high linkage disequilibrium (LD). The SNP-based approach, however, might be more strongly affected by base-to-base variation in coverage and sequencing errors. Moreover, it assumes that SNPs are independent. While this assumption might be somewhat unrealistic, natural populations of *D. melanogaster* are known to exhibit low levels of LD, with most high-level LD occurring on a scale of < 200 bp (Miyashita & Langley 1988; Langley *et al*. 2000; Mackay *et al*. 2012).

We implemented a number of stringent criteria to define alleles for analysis. We excluded all sites with a coverage < 10, since such sites are likely associated with little statistical power to identify differentiation, as well those falling within the top 2% of maximum coverage (i.e. excluding positions with > 77 reads for Florida, > 70 for Pennsylvania and > 81 for Maine), because such sites might represent copy number variants rather than true SNPs. To minimize the impact of sequencing errors and maximize the probability of calling true SNPs, we pooled counts across all populations for each position and only considered those with a minimum allele count ≥ 6 (i.e. a minimum count of two per population on average) as polymorphic; we thus assume that alleles present at high number in at least one population or occurring at low number in multiple populations represent correctly called SNPs. For most analyses, we excluded gene-poor telomeric and centromeric regions with low or no recombination since such regions are expected to yield little insight into patterns of genic differentiation; we therefore focused on the following normally recombining regions of the genome: *X*, 1,036,552-20,902,578; *2L*, 844,225-19,946,732; *2R*, 6,063,980-20,322,335; *3L*, 447,386-18,392,988; *3R*, 7,940,899-27,237,549 (Kolaczkowski *et al*. 2011).

### Estimation of population genetic parameters

To characterize genome-wide patterns of variation and differentiation, we estimated four standard population genetic parameters, *π*, Watterson's *θ*, Tajima's *D* and *F*_ST_ (Charlesworth & Charlesworth 2010). We used PoPoolation (Kofler *et al*. 2011a) to estimate *π*, *θ*_*W*_ and *D* and PoPoolation2 (Kofler *et al*. 2011b) to estimate *F*_ST_ for each pairwise population comparison (FM, FP, PM) and variable site in the genome. To estimate *π* and *θ*_*W,*_ we assumed a minimum count of two and used unbiased estimators for pooled data that correct for pool size and coverage (Futschik & Schlötterer 2010; Kofler *et al*. 2011a). Since *D* is sensitive to variation in coverage, partially due to sequencing errors, we estimated *D* by subsampling all reads to a coverage of 25, using a minimum count of one and a minimum quality of 20. Note that since *D* depends on coverage and window size, our analysis only allows for relative comparisons among our populations, not for direct comparisons with other studies. For graphical representation, we calculated average values for all statistics in nonoverlapping 200-kb windows across the entire genome (i.e. not excluding regions of low recombination). For each population or pairwise comparison, we tested for significant variation in average *π*, *θ*_*W*_ and *F*_ST_ among chromosomal arms and among populations/pairs by using two-way anova on rank-transformed means of SNP-wise values. We did not fit the interaction term since anova applied to rank-transformed data is inappropriate for interpreting interactions (Quinn & Keough 2002). To test for significance between levels of each factor, we used Tukey's HSD *post hoc* test. Since our tests for variation in *π* and *θ*_*W*_ among populations were based on ranks of means and only three populations, they were not very powerful. To further probe whether populations might differ in *π* and *θ*_*W,*_ we therefore used Kruskal–Wallis rank sum tests on the ranks of *π* and *θ*_*W*_ values estimated in nonoverlapping 200-kb windows across the entire genome. Similarly, we tested for variation in *D* among populations by using a Kruskal–Wallis rank sum test on rank-transformed *D* values estimated in 200-kb nonoverlapping windows. To identify which population pairs differ from each other in these Kruskal–Wallis analyses we used Wilcoxon rank sum *post hoc* tests.

### Identification of candidate genes

To identify genes likely to be differentiated as a result of either direct selection or indirect selection due to linkage, we used a two-pronged approach. First, to identify the most strongly differentiated alleles, we estimated pairwise *F*_ST_ for each polymorphic SNP and subjected estimates to an empirical outlier approach (Akey *et al*. 2010; Kolaczkowski *et al*. 2011). Only SNPs falling into the upper 0.5% tail of the *F*_ST_ distribution were considered to represent truly differentiated alleles at candidate loci, representing 5‰ of all SNPs found in the euchromatic, normally recombining genome. However, while this extreme value approach maximizes 'signal strength', it has two potential drawbacks: standard errors of allele frequency estimates may be highly variable because of variable sequencing coverage and testing the statistical significance of *F*_ST_ values typically requires a biologically realistic null model that can be difficult to define. Second, we therefore subjected allele counts of SNPs in the top 0.5% of the *F*_ST_ distribution to two-sided Fisher's exact tests (FET), thereby conditioning *F*_ST_ outliers on statistical significance. We only considered biallelic SNPs for FET; for multiallelic SNPs, only the two most frequent alleles were used. Since we performed a large number of tests, likely resulting in many false positives, we obtained a false discovery rate (FDR) by calculating adjusted *P*-values (*q*-values) (Storey & Tibshirani 2003) for all polymorphic sites using the LBE package in R (Dalmasso *et al*. 2005). Only SNPs with *q* < 0.01 were considered to be differentiated for our analysis. Positively identified SNPs were annotated with snpEff v2.0.3 (http://snpeff.sourceforge.net) based on reference genome r5.40 and assigned to candidate genes (±1-kb up- and downstream). While our approach is likely to miss potentially interesting candidates (Teshima *et al*. 2006), we can be quite confident about positively identified candidates.

To explore whether our set of candidates is robust, we also used an alternative method, similar to the window-based approach employed by Kolaczkowski *et al*. (2011), but based on genes rather than nonoverlapping 1-kb windows. For each gene (as defined by 5′ and 3′ UTRs plus 1-kb up- and downstream), we estimated *average F*_ST_ across *all* polymorphic SNPs (i.e. implicitly accounting for LD) and only considered those falling into the upper 5% tail of the distribution to be truly differentiated. Based on this analysis, we calculated the percentage of overlap between SNP- and gene-defined lists of candidate genes for each pairwise comparison (i.e. the overlap of the number of SNP-defined and gene-defined candidate genes divided by the number of gene-defined candidate genes).

We also investigated the size of the genomic regions differentiated between populations. We reasoned that if there is strong differentiation in the vicinity of candidate SNPs, for example due to haplotype structure, we should see a marked increase in the statistical significance of SNPs flanking the candidate SNPs. In contrast, if windows of differentiation around candidates are relatively small, we would expect that significance levels of SNPs flanking candidate SNPs decay rapidly with increasing distance from the candidates. To test this prediction, we calculated median −log_10_(*P*)-values for all flanking SNPs (including noncandidate and candidate SNPs) occurring in 100-bp windows around each candidate SNP, covering a region of 100-kb up- and downstream of candidates. To visualize potential short-range effects, we repeated the same analysis by using a higher resolution, that is, using 10-bp windows and covering a region of 500-bp up- and downstream of candidates. Median −log_10_(*P*)-values were plotted as a function of the relative distance of flanking to candidate SNPs, with the position of each candidate SNP set to zero. Note that at position zero, the median −log_10_(*P*)-value of each candidate SNP was excluded. To generate a null expectation, we estimated *P*-value levels for a random set of noncandidate SNPs ≥ 500 kb away from candidate SNPs (using the same number of SNPs and from the same chromosomal arm as the candidate SNPs) and repeated the analysis described above.

### Genome annotations

To investigate genic differentiation across genome features, we obtained *D. melanogaster* genome annotations from FlyBase r.5.40 using snpEff v2.0.3. Genome positions were annotated as coding sequence (CDS; synonymous vs. nonsynonymous sites; using a standard eukaryote codon table in snpEff), intron, 3′- and 5′- untranslated region (UTR), 1-kb downstream, 1-kb upstream, intergenic or 'other'. We calculated the proportion of features for (i) all SNPs in the normally recombining genome and (ii) all candidate SNPs. To test for over- or underrepresentation of candidate SNPs with given features, we used *χ*^2^ tests (*α* = 0.01) on SNP counts. For plots showing candidate SNPs for specific candidate genes, we included all SNPs 1 kb up- and downstream of the gene of interest to visualize SNPs located in putative regulatory regions.

### Gene ontology analysis

To analyse the biological function of candidates, we used Gene Ontology (GO) analysis (Ashburner *et al*. 2000). One problem with traditional GO analyses is that long genes have a higher probability of containing false-positive candidate SNPs than short genes containing fewer SNPs. GO categories that on average contain longer genes might thus become spuriously overrepresented. We thus tested for FDR-corrected enrichment of GO terms using Gowinda (http://code.google.com/p/gowinda/; Kofler & Schlötterer 2012), which corrects for gene length bias using a permutation approach. We obtained *D. melanogaster* GO annotations from FlyBase r.5.40 and used the following parameters in Gowinda: 10 000 000 simulations; minimum significance = 1 and minimum number of genes per GO category = 5 (i.e. excluding GO categories with <5 annotated genes). SNPs within a region of 1-kb up- or downstream of a given gene were mapped as belonging to that gene, and overlapping genes were also considered.

### Inversion analysis

Since the major cosmopolitan inversions are thought to explain a substantial proportion of clinal variation (Krimbas & Powell 1992; De Jong & Bochdanovits 2003; Rako *et al*. 2006; Hoffmann & Weeks 2007), we examined their contribution to differentiation. To investigate differentiation in- and outside inversions, we determined approximate genomic positions of breakpoints for the four major cosmopolitan inversions, *In(2L)t, In(2R)NS, In(3L)P* and *In(3R)P*, based on their cytological breakpoints (Ashburner & Lemeunier 1976). To define inversion boundaries, we chose the most proximal breakpoint at the 5′ end and the most distal at the 3′ end. Cytological locations were converted to nucleotide positions using information obtained from FlyBase (http://flybase.org/static_pages/downloads/FB2011_09/map_conversion/genome-cyto-seq.txt).

For each population and inversion, we first tested whether the proportion of candidate SNPs differs between the inversion and the rest of the chromosomal arm using FET. Similarly, for each pairwise comparison and inversion, we used Wilcoxon rank sum tests to test for differences between the average *F*_ST_-value of a given inversion and that of the rest of the arm. In addition, we estimated among-population differences in the frequency of *In(3R)P* by examining four previously published markers: an 8-bp indel marker in *hsr-omega* (Anderson *et al*. 2003) and three SNP markers in *tolkin* (C245T, T249C, T1444C; see Matzkin *et al*. 2005). To investigate SNP markers, we extracted allele frequencies from pileup files; to examine the indel marker, we visually inspected SAM alignment files using Integrative Genomics Viewer (IGV) (Robinson *et al*. 2011).

### Clinal analysis of allele frequencies

Since clinal changes in allele frequencies might reflect spatially varying selection, we analysed how frequencies of candidate SNPs change across latitude. We calculated SNP-wise allele frequencies for each population from the synchronized pileup file, conditioning for the allele that rises in frequency from South to North. For each pairwise comparison, we estimated the slopes (*s*) of frequency change across latitude between Florida (F) and Pennsylvania (P) (*s*_*1*_) and between Pennsylvania (P) and Maine (M) (*s*_*2*_) for each candidate SNP. We then subdivided each of these three sets (FP, FM, PM) into three subsets based on the sign of the two slopes (i.e. *s*_*1,2*_ = ++, +−, or −+; giving nine sets in total). Only candidate SNPs in the three ++ subsets (i.e. one ++ set for FP, FM and PM) represent alleles whose frequencies increase consistently with latitude; we therefore combined these ++ subsets to obtain a core set containing all candidate SNPs whose frequencies change clinally across populations ('plus_plus' candidates). To characterize this core set, we performed GO analysis and plotted the frequency changes of candidate SNPs for these core clinal candidate genes against latitude.

One problem with reliably estimating allele frequencies from pooled DNA data is sufficient sequence coverage; since the binomial standard error scales with sample size, low coverage might result in estimates with large standard errors or wide confidence limits. We therefore refined the core set by estimating 95% binomial confidence limits for allele frequency estimates for each SNP showing a clinal (++) pattern, using the *F* distribution: SNPs whose confidence limits do not overlap among populations exhibit significant clinal allele frequency change across latitude. In the clinal frequency plots for candidate genes, we show trajectories of these SNPs in red, against the background of all SNPs from the clinal core set shown in black.

## Results and discussion

### Genome-wide variation and differentiation

We first characterized large-scale patterns of variation and differentiation. To examine sequence variability, we used two estimates of nucleotide diversity, *π* and *θ*_*W*_. Estimates of *π* and *θ*_*W*_, when averaged over chromosomal arms, were overall higher in Florida (*π* = 0.0061; *θ*_*W*_ = 0.0063) than in Pennsylvania and Maine (average values were identical for both populations: *π* = 0.0056; *θ*_*W*_ = 0.0058) (Tables S1–S2, Supporting information). We detected significant differences for both *π* and *θ*_*W*_ among chromosomal arms, averaged over populations, with the rank order being *2L* > *2R* = *3L* > *3R* > *X* (Tables S1–S2, Supporting information). Our results for *π* are in good agreement with those for the Australian cline, with higher nucleotide diversity at lower latitude and the least amount of diversity on the *X* (Kolaczkowski *et al*. 2011). The lower diversity in Pennsylvania and Maine as compared to Florida could, for example, be due to a lower effective population size in northern populations, possibly due to contractions of population size in winter.

**Fig 2:**
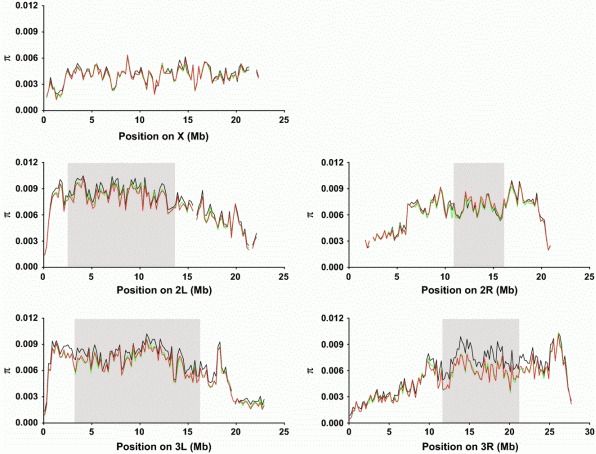
Average nucleotide diversity *π*. Average *π* across chromosomal arms, estimated over 200-kb nonoverlapping windows, shown separately for each population. Florida, black line; Pennsylvania, green line; Maine, red line. Regions with a broken line represent windows where coverage was outside the predefined minimum/maximum coverage interval, that is, windows with <60% of SNPs fulfilling the coverage criteria. Grey boxes indicate approximate regions spanned by the cosmopolitan inversions on the left and right arms of chromosome 2 (*In(2L)t*; *In(2R)NS*) and chromosome 3 (*In(3L)P*; *In(3R)P*).

When estimating *π* and *θ*_*W*_ as a function of genomic position using 200-kb nonoverlapping windows, both estimators were low near centromeres (Fig.; Begun 1992; Kolaczkowski *et al*. 2011; Kofler *et al*. 2011a; Mackay *et al*. 2012). Florida showed long genomic stretches with higher variability than Pennsylvania and Maine, particularly on *2L*, *3L* and *3R*, whereas *π* and *θ*_*W*_ were lower and similar along the whole genome for Pennsylvania and Maine (Fig. 2; Fig. S2, Supporting information). Increasing base quality to a threshold of 30 and subsampling sequence reads to a uniform genome-wide coverage of 25 did not qualitatively change patterns of *π* and *θ*_*W*_ among populations (results not shown). *θ*_*W*_ remained overall higher in Florida than in Pennsylvania and Maine (not shown), suggesting that our analysis was not strongly influenced by variation in coverage or sequencing errors.

To examine deviations from neutrality, we calculated Tajima's *D* across the whole genome. For all three populations, *D* was negative, deviating from neutrality (*D* = 0). Average *D* differed significantly among all populations (Kruskal–Wallis rank sum test, *χ*^2^ = 554, d.f. = 2, *P* < 0.001; followed by pairwise Wilcoxon rank sum *post hoc* tests, details not shown); *D* was most negative for Florida, intermediate for Maine and least negative for Pennsylvania (Fig.). A consistently negative *D* suggests an excess of rare variants, which is consistent with positive or purifying selection and/or population expansion. The pronounced excess of rare alleles in Florida might also be due to admixture from African populations, for example, via the Caribbean (Caracristi & Schlötterer 2003; Yukilevich *et al*. 2010); a higher frequency of rare variants in Florida has previously been reported, for instance, for several clinally varying metabolic loci (Sezgin *et al*. 2004).

**Fig 3:**
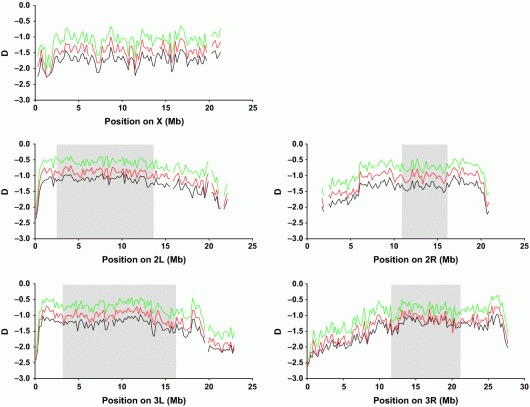
Average Tajima's *D*. Average Tajima's *D* across chromosomal arms, estimated over 200-kb nonoverlapping windows, shown separately for each population. Florida, black line; Pennsylvania, green line; Maine, red line. Regions with a broken line represent windows where coverage was outside the predefined minimum/maximum coverage interval, that is, windows with <60% of SNPs fulfilling the coverage criteria. Grey boxes indicate approximate regions spanned by the cosmopolitan inversions on the left and right arms of chromosome 2 (*In(2L)t*; *In(2R)NS*) and chromosome 3 (*In(3L)P*; *In(3R)P*).

**Fig 4:**
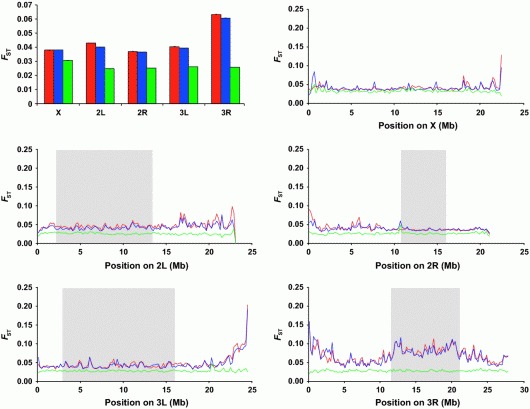
Average pairwise *F*_ST_. Upper left: Average *F*_ST_ per chromosomal arm for all polymorphic SNPs. Error bars represent standard errors of the mean (SE); error bars are typically too small to be visible. The remaining line graphs show average *F*_ST_ for each chromosomal arm (*X, 2L, 2R, 3L, 3R*), estimated over 200-kb nonoverlapping windows. Red lines: pairwise comparison Florida – Maine; blue: Florida – Pennsylvania; green: Pennsylvania – Maine. Grey boxes indicate approximate regions spanned by the cosmopolitan inversions on the left and right arms of chromosome 2 (*In(2L)t*; *In(2R)NS*) and chromosome 3 (*In(3L)P*; *In(3R)P*).

To investigate genetic differentiation among populations, we estimated pairwise *F*_ST_ for all polymorphic sites (Fig.; Table S3, Supporting information). As expected, differentiation between Florida and Maine (FM) (mean *F*_ST_ = 0.044) and between Florida and Pennsylvania (FP) (mean *F*_ST_ = 0.043) was much larger than between Pennsylvania and Maine (PM) (mean *F*_ST_ = 0.027). While mean *F*_ST_ was not significantly different between FM and FP, the amount of differentiation differed markedly between FM/FP and PM. Major differentiation between Florida and the other populations was observed on a genome-wide level and for each chromosomal arm (Fig. 4; Table S3, Supporting information). Chromosomal arm *3R* was the most strongly differentiated region of the genome between Florida and the two other populations, especially within the region of *In(3R)P* (Fig. 4). This pattern is qualitatively identical to that found by Kolaczkowski *et al*. (2011) for the Australian cline, implying a major role of *3R* and *In(3R)P* in latitudinal differentiation. In contrast to the strong differentiation seen for FM and FP, *F*_ST_ values were much smaller and similarly sized for PM, with the *X* chromosome being the most differentiated (Fig. 4, upper left panel). These findings demonstrate major latitudinal differentiation between Florida and Pennsylvania/Maine at a large number of sites spread throughout the genome, with a particular strong contribution of *3R*. Differentiation between Pennsylvania and Maine, however, was much smaller, possibly due to higher gene flow and/or similar selection pressures acting on variants shared between these populations. Interestingly, these patterns might suggest a potential disconnect between global allele frequency differentiation and phenotypic differentiation. Populations from Pennsylvania are phenotypically intermediate between those from Florida and Maine with regard to major life history traits (Schmidt & Paaby 2008), yet this apparently needs not be reflected in global sequence differentiation. While this is an interesting observation, it is currently difficult to interpret without further phenotypic and genomic data from additional populations.

### Variation and differentiation in cosmopolitan inversions

Polymorphic inversions are very common in *D. melanogaster* (Ashburner & Lemeunier 1976; Lemeunier & Aulard 1992). Previous studies have found that the four large cosmopolitan paracentric inversions (*In(2L)t, In(2R)NS, In(3L)P*, *In(3R)P*) exhibit strongly clinal patterns, for example in North America (Mettler *et al*. 1977; Knibb 1982) and Australia (Knibb *et al*. 1981), with inversion frequency being higher at lower latitudes. This pattern repeated across different geographical areas suggests that climatic selection maintains inversion frequencies (Knibb *et al*. 1981; Krimbas & Powell 1992; Hoffmann *et al*. 2004). In particular, *In(3R)Payne* is thought to be a major driver of genetic and phenotypic differentiation along latitudinal clines (Gockel *et al*. 2002; Weeks *et al*. 2002; Calboli *et al*. 2003; De Jong & Bochdanovits 2003; Kennington *et al*. 2006, 2007; Rako *et al*. 2006, 2009; Hoffmann & Weeks 2007).

We found multiple lines of evidence for a strongly clinal distribution of *In(3R)P*. First, we used four molecular markers to estimate the frequency of *In(3R)P* in each population and observed that it segregates at a frequency ≥ 0.5 (median across all four markers) in Florida but that it is almost absent in Pennsylvania (median < 0.05) and Maine (median = 0.05) (Mettler *et al*. 1977; Knibb 1982). While frequency estimates differed among markers within a given population (e.g. for Maine marker frequencies ranged from 0.02 to 0.2; FET: *P* = 0.021), our data qualitatively confirm that *In(3R)P* is much rarer (or possibly absent) at higher as compared to lower latitudes. Second, the region spanned by *In(3R)P* was significantly more differentiated than the rest of *3R* for FM and FP but not for PM, as expected from our inversion frequency estimates (Table S4, Supporting information). Thus, *In(3R)P* has a major impact on differentiation between Florida and Pennsylvania/Maine, a result that parallels the findings of Kolaczkowski *et al*. (2011) for the endpoints of the Australian cline. Third, within the region spanned by *In(3R)P,* average *π* was significantly higher in Florida (*π* = 0.0077) as compared to Pennsylvania (*π* = 0.0061) and Maine (*π* = 0.0061) (Wilcoxon rank sum test, both cases: *P* < 0.001), whereas *π* did not differ between Pennsylvania and Maine (*P* = 0.94) (also see Fig. 2).

The other inversions showed much less clear effects on differentiation than *In(3R)P* (Table S4, Supporting information). For FM and FP, median *F*_ST_ values were significantly lower within *In(2L)t* and *In(2R)NS* and not different within *In(3L)P* as compared to the rest of the chromosomal arms. However, for PM the median *F*_ST_ within *In(2L)t* was significantly higher than for the rest of *2L*. While we did not investigate the frequencies of major cosmopolitan inversions other than *In(3R)P*, the frequencies of *In(2L)t, In(2R)NS* and *In(3L)P* are also known to vary strongly clinally along the east coast of the United States (Mettler *et al*. 1977; Knibb 1982). Thus, even though the frequencies of these inversions likely differ between our populations, we failed to observe major differentiation in the regions spanned by them.

### Genic patterns of population differentiation

**Fig 5:**
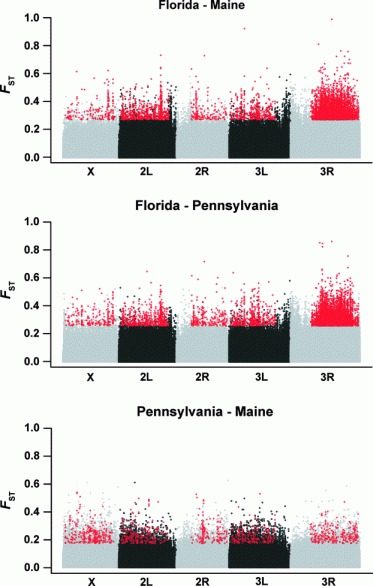
Pairwise *F*_ST_ of candidate SNPs. Pairwise *F*_ST_ values of all candidate SNPs for each comparison (top: Florida – Maine; centre: Florida – Pennsylvania; bottom: Pennsylvania – Maine). Different chromosomal arms are indicated by alternating grey and black; noncandidate SNPs are shown as grey or black circles; candidate SNPs are shown as red circles. Plots include low- and nonrecombining regions; this can be seen by some grey and black circles representing noncandidate SNPs with high *F*_ST_ values – these might for instance represent copy number variants.

We next identified and characterized candidate genes that underlie population differentiation. In total, our data contained ~1.5 million polymorphic SNPs in 11 314 genes. After excluding sites with low recombination, we defined candidate genes as those that contained SNPs whose *F*_ST_-values fell into the top 0.5% of the *F*_ST_ distribution and that showed statistically significant allele frequency differentiation among populations after FDR correction (*q* < 0.01). We identified 12 090 candidate SNPs in 3169 candidate genes across all three pairwise comparisons; for FM we found 6673 candidate SNPs in 2010 candidate genes, for FP 6892 in 2051 and for PM 1149 in 720 (Fig. S4, Table S5, Supporting information). *F*_ST_ scaled well with geographical distance between populations, with both average and maximum *F*_ST_ being highest for FM, slightly lower for FP and lowest for PM (Table 1, Fig. 5). As expected, FM and FP showed substantial overlap in the number of shared candidate genes (1109), suggesting that we successfully identified putative targets of selection consistently differentiated between Florida and Pennsylvania/Maine and reflecting low differentiation for PM (Fig. S4, Supporting information; Table 1). In contrast, PM only shared 243 candidate genes with FM and 260 with FP. Consequently, we found a relatively small number of candidate genes (160) shared among all three comparisons (Fig. S4, Supporting information; Table 1). A likely explanation is that the amount of differentiation between Pennsylvania and Maine is very small relative to FM and FP (Fig. 5). This might be because the frequency of *In(3R)P*, which harbours a large number of candidates, decreased substantially from Florida to Pennsylvania/Maine, whereas its frequency was very small (possibly zero) in Pennsylvania and Maine and practically indistinguishable between these two populations (also see Mettler *et al*. 1977; Knibb 1982).

**Table 1:** Average pairwise *F*_ST_ values of candidate SNPs. Pairwise comparisons: FM, Florida – Maine; FP, Florida – Pennsylvania; PM, Pennsylvania – Maine. SE, standard error of the mean. *q*-Values from FET on allele frequencies

Comparison	Mean *F*_ST_	SE	Range	−(log_10_) *q*-value range	No. of candidates
FM	0.34	0.0007	0.28–1	2.00–15.28	6673
FP	0.33	0.0007	0.27–0.87	2.00–16.04	6892
PM	0.24	0.0018	0.17–0.54	2.00–8.74	1149

Candidates for latitudinal differentiation were enriched on *3R* for both FM and FP (77% of all candidate SNPs for both comparisons; FM: 5114 SNPs in 1218 genes; FP: 5286 SNPs in 1178 genes; *χ*^2^ test: *P* < 0.001) but underrepresented on all other chromosomal arms as compared to genomic background (*χ*^2^ test: *P* < 0.001) (Fig. 5). Within *3R*, candidates for FM and FP were enriched in *In(3R)P* as compared to the rest of the arm (Table S6, Supporting information), with more than 50% of all FM and FP candidate SNPs occurring in this inversion (FM: 51%; 3425 SNPs in 735 genes; FP: 52%; 3597 SNPs in 737 genes; Fig. 5), underscoring the major role of *3R* and *In(3R)P* in shaping latitudinal differentiation. Candidates for FM and FP were also overrepresented within the region of *In(3L)P*, an inversion whose frequency is negatively correlated with cold resistance (Weeks *et al*. 2002), but underrepresented within *In(2L)t* and *In(2R)NS* (Table S6, Supporting information; Fig. 5). In contrast, most candidates for PM were located on the *X* (26% of all candidate SNPs; 299 SNPs in 162 genes; *χ*^2^ test: *P* < 0.001), whereas candidates were underrepresented on *2L*, *2R* and *3L* (*χ*^2^ tests: all *P* < 0.01). Notably, for PM, we failed to find enrichment of candidates on *3R* and within *In(3R)P* as compared to the rest of *3R*, with only 9% (107 SNPs in 82 genes) of all candidates occurring in this inversion (Table S6, Supporting information; Fig. 5). This confirms that differentiation between Pennsylvania and Maine is largely independent of *In(3R)P*, in agreement with our inversion frequency estimates. Only 377 genes were differentiated between Pennsylvania and Maine, thus representing candidates that might be independent of *In(3R)P* (Fig. S4, Table S5, Supporting information). This small number of candidates might indicate that only few genes within the region of *In(3R)P* are locally adapted and that most of the elevated differentiation in this region is due to linkage within the inversion. Interestingly, candidates within the region spanned by *In(2R)NS* were underrepresented for all pairwise comparisons, suggesting a consistent deficiency of genes contributing to differentiation across populations in this inversion, although it is known to harbour several clinal loci (Lemeunier & Aulard 1992).

Since our SNP-based candidate gene approach rests on the somewhat unrealistic assumption that SNPs are independent (no LD), we tested the robustness of our method by using a gene-based approach, similar to the window-based method used by Kolaczkowski *et al*. (2011). We estimated average *F*_ST_ across all polymorphic sites within a given gene for each pairwise comparison and defined candidate genes as those with an average *F*_ST_ in the top 5% of the distribution. When applied to this set, our SNP-based approach detected 86% of all candidate genes for FM, 88% for FP and 22% for PM (details not shown), indicating that both methods yield largely similar results, at least for FM and FP. The rather small overlap between the methods for PM might reflect the small amount of differentiation between Pennsylvania and Maine; since effect sizes of allele frequency differences for PM were small, the SNP-based approach might be much more conservative when applied to PM than the gene-based approach which does not condition *F*_ST_ values on significant FET. Interestingly, when we excluded candidates on *3R*, the overlap between the two approaches decreased to 63% (−25%) for FM and to 67% (−21%) for FP, but increased to 27% (+5%) for PM. In general, we favour using the SNP-based over the gene- or window-based approach, especially when differentiation between populations is not very large.

Next, we investigated the size of genomic regions differentiated between populations. We predicted that strong differentiation in the neighbourhood of candidate SNPs, for instance due to haplotype structure, would elevate the statistical significance of SNPs flanking candidate SNPs, resulting in broad peaks around candidates. In all pairwise comparisons, and for all chromosomal arms except *3R*, −log_10_(*P*)-values rapidly dropped to random background levels within ~100 bp (Fig. S5A–B, Supporting information). In contrast, for FM and FP, −log_10_(*P*)-values decayed more slowly on *3R* as compared to other chromosomal arms, resulting in a broad base extending over > 500 bp up- and downstream of candidates, with −log_10_(*P*)-values converging asymptotically to random background (Fig. S5B, Supporting information). To examine whether this was caused by *In(3R)P,* we asked whether the −log_10_(*P*)-value distributions differ between the inversion and the rest of *3R* (Fig. S5C–D, Supporting information). Within the inversion, −log_10_(*P*)-values were on average higher, both for the baseline and random background, than outside the inversion (Fig. S5C, Supporting information). Our results thus suggest an excess of differentiated variants on this chromosomal arm. However, when averaging −log_10_(*P*)-values for each pairwise comparison across all autosomes and excluding *3R*, −log_10_(*P*)-values still decayed much more slowly to background for FM and FP than for PM (Fig. S5E, Supporting information). Thus, Florida appears to exhibit generally more differentiation than the other two populations independent of *3R* and *In(3R)P*.

### Biological description of candidate genes

To biologically characterize our candidate genes, we first examined differentiation of candidates across genome annotations (Table S7, Supporting information). Nonsynonymous sites were overrepresented in both FM (4%; *χ*^2^ test: *P* = 0.002) and FP (3%; *χ*^2^ test: *P* = 0.007) but not in PM (Table 2), indicating selection at the protein level between Florida and Pennsylvania/Maine. This contrasts with the results of Kolaczkowski *et al*. (2011) who did not find overrepresentation of protein-coding sequence in their data for the Australian cline (also see discussion below). Two interesting examples of strong nonsynonymous differentiation are the immunity genes *Helicase89B (Hel89B)*, which positively regulates expression of antimicrobial peptides (Yagi & Ip 2005), and *immune-regulated catalase* (*Irc*), which is required in the gastrointestinal tract during host–microbe interactions (Ha *et al*. 2005a,b) and which also shows nonsynonymous differentiation along the Australian cline (Kolaczkowski *et al*. 2011). Given that North American populations vary clinally in egg production (Schmidt *et al*. 2005a,b; Schmidt & Paaby 2008), another interesting candidate showing nonsynonymous differentiation is *twin*, a gene important for germ line cyst development and oocyte fate (Morris *et al*. 2005). Numerous other examples of nonsynonymous differentiation can be found in Table S7 (Supporting information). Notably, while synonymous changes are classically assumed to be neutral, we found that synonymous sites were enriched for FM and FP but underrepresented for PM (Table 2; Table S7, Supporting information). Although the significance of this pattern remains unclear, several studies have shown that selection can act on synonymous sites, for example affecting translational efficiency or thermodynamic stability of mRNA (Shields *et al*. 1988; Cuevas et al. 2011). It also remains possible that the differentiation we have observed at synonymous sites is caused by linkage within genes that are targets of selection. Thus, our data suggest that 'silent' variants might play a role in latitudinal differentiation, although we cannot conclusively say whether this pattern is due to demography or selection. Unlike Kolaczkowski et al. (2011), we failed to detect over- or underrepresentation of 5'- and 3'-UTRs, but regions 1 kb downstream of candidate genes were enriched for FM and FP, possibly due to regulatory polymorphisms in these regions. Moreover, intergenic regions were underrepresented for FM and FP (Table 2; Table S7, Supporting information). For reasons presently unclear , our findings on differentiation across genome annotations do not agree particularly well with those of Kolaczkowski *et al*. (2011). One possibility might be that this discrepancy is due to the different timescales of differentiation for the Australian and North American cline, with *D. melanogaster* having colonized North America most likely prior to 1875 (Keller 2007), whereas the Australian cline is only about 100 years old (Hoffmann & Weeks 2007). This might, for example, explain differences among the two clines in the availability of nonsynonymous coding sequence variants which are expected to be much less polymorphic and available to selection on standing variation than synonymous variants during initial colonization and establishment of the cline (see discussion in Kolaczkowski *et al*. 2011).

**Table 2:** Differentiation of candidate SNPs across genome annotations. Numbers are proportions of candidate SNPs with a particular feature; proportions were tested using *χ*^2^ tests, with significant (*α* = 0.01) over- or underrepresentation shown in boldface (ov, overrepresented; un, underrepresented). Pairwise comparisons: FM, Florida – Maine; FP, Florida – Pennsylvania; PM, Pennsylvania – Maine

Feature	FM	FP	PM
Downstream	**0.12**^**ov**^	**0.12**^**ov**^	0.10
Intergenic	**0.14**^**un**^	**0.14**^**un**^	0.19
Intron	0.38	0.37	0.40
Nonsynonymous coding	**0.04**^**ov**^	**0.03**^**ov**^	0.02
Synonymous coding	**0.13**^**ov**^	**0.14**^**ov**^	**0.07**^**un**^
Upstream	0.13	0.14	0.16
3′UTR	0.02	0.03	0.02
5′UTR	0.02	0.02	0.02
Other	0.004	**0.005**^**ov**^	0.003

To further characterize candidates, we performed GO analysis with Gowinda (Table S8, Supporting information) but failed to find significant enrichment of GO terms after FDR correction. Since Gowinda corrects for gene length bias by assuming complete linkage of SNPs within genes, power may be lower than for approaches that model the true underlying haplotype structure. For example, the top three GO categories with the lowest *P*-values had FDR-values between 0.19 and 0.22 for FM, 0.17 for FP and between 0.16 and 0.30 for PM. Although not being significant at a standard FDR threshold (e.g. FDR = 0.05), it is noteworthy that the top three categories for FM and FP were all related to metabolism (FM: 'metabolic process', 'proline metabolic process', 'primary metabolic process'; FP: 'proline metabolic process', 'protein metabolic process' and 'metabolic process'), whereas for PM the top three categories were all related to pathogen defence and immunity ('antibacterial humoural response', 'defence response', 'response to bacterium'). Despite the lack of significance, these patterns are consistent with those reported by Turner *et al*. (2008) and Kolaczkowski *et al*. (2011) who also found enrichment for GO terms related to metabolism and immunity; however, in contrast to our GO analysis, these studies did not correct for gene length bias. Importantly, when we performed GO analysis without correcting for gene length bias, we detected significant enrichment in dozens of GO categories (not shown), similar to Turner *et al*. (2008) and Kolaczkowski *et al*. (2011). Thus, in contrast to the commonly held view that significant GO enrichment might be indicative of selection, our results suggest that in the absence of gene length correction many GO patterns might be spurious and can therefore not necessarily be taken as strong evidence for spatially varying selection.

**Fig 6:**
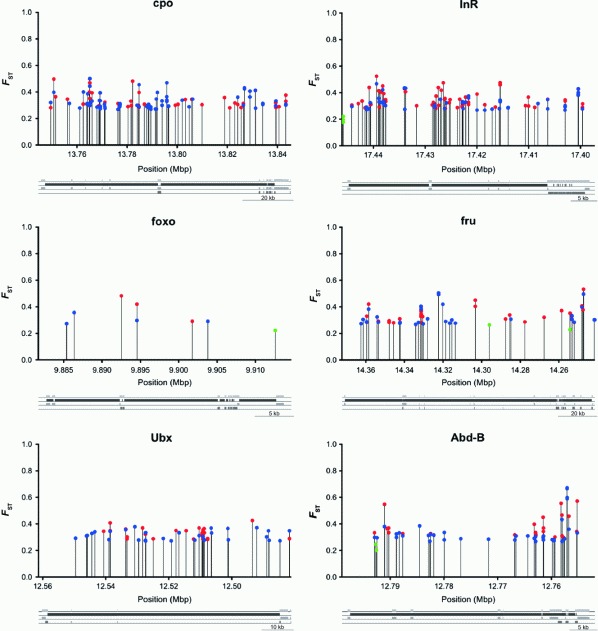
Examples of latitudinally differentiated candidate genes. *F*_ST_ of candidate SNPs in six exemplary candidate genes. The two genes shown in the top row (couch potato, cpo*,* and Insulin-like receptor, InR) have been previously found to vary clinally, whereas the remaining four genes (forkhead-box subgroup O, foxo; fruitless, fru; Ultrabithorax, Ubx*,* and Abdominal B, Abd-B) are novel candidates. Gene maps indicate locations of exons (first line from top), introns (second line), untranslated regions (UTRs; third line) and coding sequences (CDS; fourth line, bottom). For clarity, only one isoform is shown for genes that have multiple isoforms. Red dots: Florida – Maine; blue: Florida – Pennsylvania; green: Pennsylvania – Maine.

To supplement our analysis of candidates, we hand-curated functional information from FlyBase and the literature. Although candidates did not fall into significantly enriched GO categories, we identified hundreds of strongly differentiated genes in major functional pathways (see Table S5, Supporting information). Notably, our results not only identify numerous novel candidates but also confirm many genes and pathways previously implicated in latitudinal differentiation (also see Turner *et al*. 2008; Kolaczkowski *et al*. 2011). Specifically, we found that 644 candidate genes differentiated between the endpoints of the US cline (FM) were also significantly differentiated between the endpoints of the Australian cline (Queensland vs. Tasmania; see Kolaczkowski *et al*. 2011), which corresponds to a significant overlap of 31% between these candidate sets as compared to random expectation (*P* < 0.0001; see Table S9, Supporting information). Thus, while we cannot rule out that some of our candidates are false positives, and although we cannot formally prove that these loci are under selection, the major overlap with previously and independently identified candidates strongly suggests that our candidate genes represent targets of spatially varying selection and that differentiation at these loci is unlikely due to demography alone. Figure 6 shows patterns of *F*_ST_ differentiation for candidate SNPs in six exemplary candidate genes; Appendix S1–S12 (Supporting information) show examples of candidate genes in major biological pathways, including hand-curated functional information from FlyBase and the literature. For a full list of candidate genes, see Table S5 (Supporting information).

Many candidate genes have known roles in life history regulation (Flatt & Heyland 2011), and it is thus tempting to hypothesize that natural variants at these loci might underlie latitudinal differentiation in fitness-related traits. Notably, hormones are critical physiological regulators of life history traits (Finch & Rose 1995; Flatt & Heyland 2011), and we found many of our candidates to be involved in hormone signalling and production (Appendix S1–S3, Supporting information). In the insulin/insulin-like growth factor (IIS) and target of rapamycin (TOR) pathways, important for regulating growth, body size, metabolism, reproduction and lifespan (Oldham & Hafen 2003; Tatar *et al*. 2003), we found strongly differentiated SNPs in numerous genes, for example in two *Drosophila insulin-like peptides* (*dilps 3* and *5*); the *insulin-like receptor* (*InR*), previously found to vary clinally and affect life history traits in natural populations (Paaby *et al*. 2010; Kolaczkowski *et al*. 2011; Fig. 6); *phosphatidylinositol-4,5-bisphosphate 3-kinase* (*Pi3K*), previously linked to natural variation in reproductive dormancy (Williams *et al*. 2006); the forkhead transcription factor *foxo* downstream of IIS (Fig. 6) and in *target of rapamycin* (*Tor*) (Appendix S1, Supporting information). These findings are interesting in view of the fact that genetic manipulations of IIS/TOR are known to have major effects on life history traits in the laboratory (Tatar *et al*. 2003; Giannakou & Partridge 2007); in particular, they are consistent with the hypothesis that clinal variation in life history traits, for example body size, might be driven by natural variation in IIS/TOR signalling (De Jong & Bochdanovits 2003).

We also detected at least 14 candidate genes involved in ecdysone signalling and production (Appendix S2, Supporting information), a pathway important for regulating larval growth, body size, metamorphosis, ovarian development, reproductive dormancy, lifespan and immune function (Kozlova & Thummel 2000; Flatt *et al*. 2008; Galikova *et al*. 2011; Schmidt 2011). The perhaps most interesting candidate in this pathway is *couch potato* (*cpo*), a gene that is expressed in several tissues including the ring gland (larval site of ecdysone production), contains a large number of ecdysone response elements, varies both along the North American and Australian cline and underlies natural clinal variation in reproductive dormancy along the US east coast (Schmidt *et al*. 2008; Kolaczkowski *et al*. 2011; Schmidt 2011) (Fig. 6). Six of our candidates (*cpo*; the ecdysone inducible proteins *Eip63E*, *Eip74EF*, *Eip75B*, *Eip93F*; and *Samuel*) were also found for the Australian cline (Kolaczkowski *et al*. 2011) and three (*Eip63E*, *Eip74EF*, *Eip75B*) in an artificial selection experiment on body size (Turner *et al*. 2011), a trait known to vary clinally (David & Bocquet 1975; De Jong & Bochdanovits 2003). Moreover, a recent genomic study of latitudinal differentiation in *Anopheles gambiae* also detected strong differentiation in this pathway (Cheng *et al*. 2012). In contrast to Kolaczkowski *et al*. (2011), however, we did not detect significant differentiation at the *ecdysone receptor* (*EcR*) locus. Several nuclear hormone receptor and other endocrine genes, some of which are known to interact with ecdysone signalling (King-Jones & Thummel 2005), were also differentiated, including *eclosion triggering hormone receptor* (*ETHR*) (Appendix S3, Supporting information). Interestingly, Kolaczkowski *et al*. (2011) failed to find differentiation in *ETHR* but found clinal variation at *eclosion hormone* (*Eh*), the gene encoding the ligand for this receptor. The fact that these endocrine pathways all have major metabolic functions is perhaps consistent with the observation that the top three GO categories for FM and FP are related to metabolism (Table S8, Supporting information). In line with this, we also found several genes involved in lipid metabolism to be differentiated (Appendix S4, Supporting information).

Genes in the Toll/Imd pathways, involved in the regulation of innate immunity (Hoffmann 2003; Ferrandon *et al*. 2007), represent another major class of candidates (Appendix S5, Supporting information). Strongly differentiated candidates included *peptidoglycan recognition proteins* (*PGRPs*), central signalling components, such as *immune deficiency* (*imd*) and *Toll*, and various antimicrobial peptides such as *Diptericin* (*Dpt*) and *Drosocin* (*Dro*). Kolaczkowski *et al*. (2011) also observed enrichment of candidates in the Toll pathway as well as differentiation in other immunity genes, such as *Irc* and *sick* (*sickie*), which we also found. Our data thus indicate that latitudinal adaptation involves strong spatially varying selection on immunity, possibly due to variation in pathogen diversity and abundance across latitude (also see Turner *et al*. 2008; Kolaczkowski *et al*. 2011). In support of this notion, immunity genes are known to harbour a lot of genetic variation and to be under strong selection in natural populations (Lesser *et al*. 2006; Lazzaro 2008).

Several other central *Drosophila* signalling pathways contained differentiated candidate genes in our data, including EGFR, JAK/STAT, TGF-β/BMP and torso signalling; certain members of these pathways are known regulators of growth, body size, metamorphosis, reproductive development, immunity and metabolism (Appendix S6–S9, Supporting information). Again, we found differentiation in many candidates in these pathways also identified by Kolaczkowski *et al*. (2011), confirming that they are important targets of clinal selection. Similarly, clinal differentiation of candidates in the EGFR and TGF-β/BMP pathways has also been found in *A. gambiae* (Cheng *et al*. 2012). Genes involved in the molecular regulation of circadian rhythms were differentiated as well, including *timeless* (*tim*), *timeout* and *cryptochrome* (*cry*), which have all previously been found to vary clinally (Sandrelli *et al*. 2007; Tauber *et al*. 2007; Turner *et al*. 2008; Kolaczkowski *et al*. 2011), as well as a novel clinal candidate, *clock* (*Clk*) (Appendix S10, Supporting information). Yet, unlike other studies (Costa *et al*. 1992; Sawyer *et al*. 1997; Turner *et al*. 2008; Kolaczkowski *et al*. 2011), we failed to find differentiation in the *period* (*per*) locus, presumably due to our rather stringent criteria for defining candidates (see details in Appendix S10, Supporting information). Differentiation in this pathway is noteworthy because it has been implicated in the photoperiodic regulation of reproductive dormancy (Sandrelli *et al*. 2007; Tauber *et al*. 2007), which is known to vary clinally (Schmidt *et al*. 2005a,b; Schmidt & Paaby 2008). We also observed differentiation in several candidates involved in learning and memory (Appendix S11, Supporting information). One of the most prominent genes in this group is *foraging* (*for*), a cGMP-dependent protein kinase known to harbour a natural larval behavioural polymorphism (Osborne *et al*. 1997), which also affects adult learning and memory (Mery *et al*. 2007). Interestingly, *for* is also involved in the metabolic response to food deprivation by interacting with IIS (Kent *et al*. 2009). This locus was also found to be differentiated by Turner *et al*. (2008), and Kolaczkowski *et al*. (2011) similarly found enrichment of candidates involved in the development of the mushroom bodies, brain structures important for learning and memory. Finally, several well-known transcription factor genes showed clear patterns of differentiation, for example *Ultrabithorax* (*Ubx*) and *Abdominal-B* (*Abd-B*), two major *Hox* genes critically important for development, as well as *fruitless* (*fru*), a gene involved in determining sex-specific mating behaviour (Appendix S12, Supporting information; Fig. 6).

### Clinal allele frequency change in candidate genes

To explicitly investigate the clinal dynamics of candidate genes, we analysed how allele frequencies change with latitude. While our data allowed us to go beyond comparing the endpoints of the cline (Turner *et al*. 2008; Kolaczkowski *et al*. 2011), our analysis based on only three populations is necessarily somewhat provisional. Nonetheless, our results confirm several genes previously reported to vary clinally and reveal numerous novel clinal candidates. For a full list of these clinal candidate genes, see Appendix S14 (Supporting information); many of the biologically interesting candidates discussed above are contained in this list.

We conditioned each SNP for the allele increasing in frequency between Florida and Maine and examined frequency changes across all populations. This resulted in three possible classes of frequency change: alleles that (i) show a constant increase across all populations; (ii) first drop in frequency between Florida and Pennsylvania and then increase between Pennsylvania and Maine and (iii) first increase in frequency between Florida and Pennsylvania and then drop between Pennsylvania and Maine. The latter two classes might not necessarily reflect clinal selection but might contain genes differentiated due to local adaptation or genetic drift. We therefore only considered SNPs showing a consistent increase in frequency across all populations and merged them into one clinal data set ('plus_plus' candidates), comprising 1974 candidate genes (6117 SNPs in total; FM: 88.4% of all candidate SNPs; FP: 28.5%; PM: 6.6%). For each of these candidate genes, we show plots of allele frequency against latitude in Appendix S14 (Supporting information). Importantly, numerous of these clinal candidates have also been found by Kolaczkowski *et al*. (2011) for the Australian cline (also see Table S9, Supporting information).

To functionally characterize these clinal candidates, we performed GO analysis using Gowinda to correct for gene length bias. The top three GO categories in terms of the lowest *P*-values were 'RNA methylation' (FDR = 0.04) and, similar to our analyses for FM and FP above, 'metabolic process' (FDR = 0.12) as well as 'primary metabolic process' (FDR = 0.24) (Table S8, Supporting information). We further refined our core set of clinal candidates by restricting the analysis to those SNPs whose 95% confidence intervals for allele frequencies did not overlap among populations; such SNPs show the steepest frequency change with latitude. This yielded a set of 173 significantly clinal SNPs located in 141 candidate genes (Appendix S14, Supporting information; plots containing SNPs with trajectories in red). Since confidence limits were often quite large, and because we only had data for three populations along the cline, this is a relatively small set of clinal candidates. To our knowledge, only 13 genes (9.3%) of this set have previously been mentioned in the literature as varying clinally: *CG5466*; *CG31320*; *CG31380*; the cardioacceleratory peptide receptor *CcapR*; *cpo*; *Eip63E*; the gustatory receptor *Gr36a*; the transcription factor *Ino80*; *InR*; the lipophorin receptor *LpR1*; *sick*; the JAK/STAT transcription factor *Stat92E*; and the transglutaminase *Tg* (Schmidt *et al*. 2008; Turner *et al*. 2008; Kolaczkowski *et al*. 2011; Paaby *et al*. 2010). Although we did not perform a comprehensive comparison with published data, this suggests that most of our significantly clinal candidates are novel. GO analysis of this refined set did not yield significant results (all FDRs > 0.25; not shown), probably due to a lack of statistical power and our conservative correction for gene length bias.

**Fig 7:**
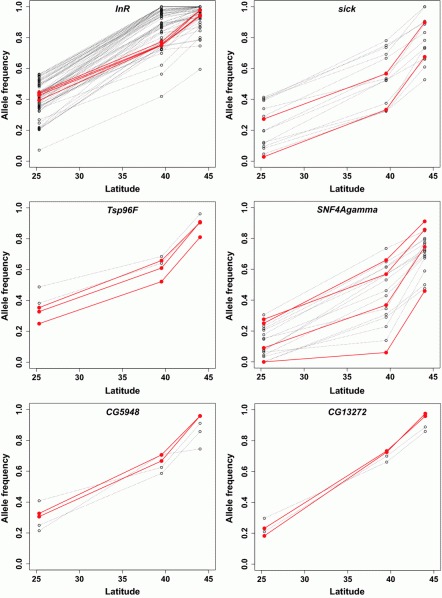
Examples of clinal SNPs in candidate genes. Allele frequencies of candidate SNPs in six exemplary candidate genes, rising in frequency across the cline, from low (Florida) to high latitude (Maine). The two genes in the top row (Insulin-like receptor, InR, and sickie*,* sick) have been previously found to vary clinally, whereas the remaining four are novel candidates (Tetraspanin 96F*,* Tsp96F*;* SNF4/AMP-activated protein kinase gamma subunit*,* SNF4Agamma; CG5948 and CG13272). Red lines indicate SNPs whose 95% binomial confidence intervals do not overlap across populations (latitudes).

Some interesting examples of clinal candidate genes and their allele frequency trajectories across latitude are shown in Fig. 7. The top two panels depict two candidates already known to vary clinally, *InR* (Paaby *et al*. 2010) and *sick* (Kolaczkowski *et al*. 2011), whereas the bottom four panels show examples of newly identified clinal candidate genes that contain SNPs whose frequencies change very strongly with latitude: *Tetraspanin96F* (*Tsp96F*), a gene with unknown molecular function and phenotypic effect; *SNF4/AMP-activated protein kinase gamma subunit* (*SNF4Agamma*), a gene involved in lipid metabolism and the response to starvation (Johnson *et al*. 2010); *CG5948*, a gene with putative, electronically inferred roles in metal ion binding, oxidation reduction and superoxide metabolism; and *CG13272*, again a gene whose function is completely unknown (see FlyBase for further information).

## Conclusions

Many previous studies have found major phenotypic and genetic differentiation in *D. melanogaster* along the well-known North American cline, a pattern thought to be caused by spatially varying selection (Oakeshott *et al*. 1982; Singh & Rhomberg 1987; Coyne & Beecham 1987; Hale & Singh 1991; Berry & Kreitman 1993; Schmidt *et al*. 2008; Turner *et al*. 2008; Paaby *et al*. 2010). Despite major progress (Turner *et al*. 2008), however, our understanding of the genetic basis of latitudinal differentiation along this cline is still limited. In an attempt to complement and extend recent genomic efforts towards understanding clinal variation in *D. melanogaster* (Turner *et al*. 2008; Kolaczkowski *et al*. 2011), we have performed the first genome-wide next-generation sequencing analysis of latitudinal differentiation along the North American cline. Our results are consistent with the hypothesis that hundreds of key genes and many important functional pathways might experience pervasive spatially varying selection along this cline, with many of the candidates being involved in the regulation of life history traits and metabolism. Our data thus provide a comprehensive catalogue of candidate genes for phenotypes known to vary clinally, including fitness-related traits such as body size, fecundity, lifespan and reproductive dormancy. Despite important limitations of the quantitative trait nucleotide (QTN) approach (Rockman 2012), it will clearly be of major interest to functionally analyse the phenotypic effects of natural variants we have uncovered. Interestingly, several of the pathways we have identified interact strongly with each other and are known to have highly pleiotropic phenotypic effects (also see Kolaczkowski *et al*. 2011), for example IIS and ecdysone signalling interact in regulating larval growth (Colombani *et al*. 2005) as well as reproductive dormancy (Schmidt 2011), and ecdysone signalling transcriptionally regulates the expression of antimicrobial peptides involved in humoral innate immunity (Flatt *et al*. 2008). If populations harbour genetic variance for such molecular interactions, genic targets of spatially varying selection might not be independent of each other. In this case, latitudinal adaptation might involve correlational selection, acting on suites of correlated phenotypes caused by genetic correlations, for example due to pleiotropy, epistasis or linkage (Sinervo & Svensson 2002). Indeed, one of our most important findings is that the majority of candidate SNPs and genes we have identified are located within the region spanned by the major cosmopolitan inversion on *3R*, *In(3R)Payne*. This striking pattern might be consistent with the idea that *In(3R)Payne* represents a 'coadapted gene complex' or 'supergene' (Dobzhansky 1970; also see Krimbas & Powell 1992; Schaeffer *et al*. 2003; Hoffmann *et al*. 2004) or, alternatively, with strong linkage and hitchhiking within this inversion. Importantly, our data also provide compelling evidence for major parallel differentiation at numerous loci between the North American and Australian clines, a pattern that is most parsimoniously explained by spatially varying selection and that is unlikely solely due to demography. While the caveat remains that we cannot conclusively prove that our candidates are subject to selection, and while demonstrating selection will require in-depth studies of individual candidate genes and QTNs, our results considerably strengthen the case for spatially varying selection across latitude at numerous loci spread throughout the genome.

## Data accessibility

DNA sequences: http://www.ebi.ac.uk/ena/data/view/ERP001535

## Supporting Information

**Fig. S1** Average coverage of pool-seq samples.

**Fig. S2** Average scaled population mutation rate *θ*_*W*_.

**Fig. S3** Frequency of the major cosmopolitan inversion *In(3R)Payne*.

**Fig. S4** Overlap of candidate genes among population comparisons.

**Fig. S5** Decay of statistical significance around candidate SNPs.

**Table S1** Average nucleotide diversity π.

**Table S2** Average scaled population mutation rate *θ*_*W*_.

**Table S3** Average pairwise *F*_ST_ values.

**Table S4** Average pairwise *F*_ST_ in- and outside inversions.

**Table S5** Top candidate genes.

**Table S6** Proportion of candidate SNPs within inversions.

**Table S7** Genome annotations of candidate genes.

**Table S8** Gene ontology (GO) analysis of candidates.

**Appendix S1** Examples of candidate genes in the insulin/insulin-like growth factor (IIS) and target of rapamycin (TOR) signaling pathways.

**Appendix S2** Examples of candidate genes involved in ecdysone biosynthesis and signaling.

**Appendix S3** Examples of candidate genes involved in nuclear hormone receptor (NHR) signaling and other endocrine pathways.

**Appendix S4** Examples of candidate genes involved in lipid metabolism.

**Appendix S5** Examples of candidate genes involved in innate immunity (Toll/Imd signaling).

**Appendix S6** Examples of candidate genes involved in epidermal growth factor (EGFR) signaling.

**Appendix S7** Examples of candidate genes involved in JAK/STAT signaling.

**Appendix S8** Examples of candidate genes involved in TGF-β/BMP signaling.

**Appendix S9** Examples of candidate genes involved in torso signaling.

**Appendix S10** Examples of candidate genes involved in circadian rhythms.

**Appendix S11** Examples of candidate genes involved in learning and memory.

**Appendix S12** Examples of some transcription factor candidate genes.

**Appendix S13** Description of the bioinformatic analysis pipeline, including Python scripts.

**Appendix S14** Plots for each clinal ('plus_plus') candidate gene, showing the changes in allele frequency for the candidate gene as a function of latitude.
